# Improving the Safety, Effectiveness, and Efficiency of Clinical Alarm Systems: Simulation-Based Usability Testing of Physiologic Monitors

**DOI:** 10.2196/20584

**Published:** 2021-02-03

**Authors:** Azizeh K Sowan, Nancy Staggers, Andrea Berndt, Tommye Austin, Charles C Reed, Ashwin Malshe, Max Kilger, Elma Fonseca, Ana Vera, Qian Chen

**Affiliations:** 1 School of Nursing University of Texas Health at San Antonio San Antonio, TX United States; 2 School of Nursing and Department of Biomedical Informatics University of Utah Salt Lake City, UT United States; 3 University Health San Antonio, TX United States; 4 College of Business University of Texas at San Antonio San Antonio, TX United States; 5 College of Electrical and Computer Engineering University of Texas at San Antonio San Antonio, TX United States

**Keywords:** usability testing, clinical alarms, fatigue, critical care, patient safety, nursing

## Abstract

**Background:**

Clinical alarm system safety is a national patient safety goal in the United States. Physiologic monitors are associated with the highest number of device alarms and alarm-related deaths. However, research involving nurses’ use of physiologic monitors is rare. Hence, the identification of critical usability issues for monitors, especially those related to patient safety, is a nursing imperative.

**Objective:**

This study examined nurses’ usability of physiologic monitors in intensive care units with respect to the effectiveness and efficiency of monitor use.

**Methods:**

In total, 30 nurses from 4 adult intensive care units completed 40 tasks in a simulation environment. The tasks were common monitoring tasks that were crucial for appropriate monitoring and safe alarm management across four categories of competencies: admitting, transferring, and discharging patients using the monitors (7 tasks); managing measurements and monitor settings (23 tasks); performing electrocardiogram (ECG) analysis (7 tasks); and troubleshooting alarm conditions (3 tasks). The nurse-monitor interaction was video-recorded. The principal investigator and two expert intensive care units nurse educators identified, classified, and validated task success (effectiveness) and the time of task completion (efficiency).

**Results:**

Among the 40 tasks, only 2 (5%) were successfully completed by all the nurses. At least 1-27 (3%-90%) nurses abandoned or did not correctly perform 38 tasks. The task with the shortest completion time was “take monitor out of standby” (mean 0:02, SD 0:01 min:s), whereas the task “record a 25 mm/s ECG strip of any of the ECG leads” had the longest completion time (mean 1:14, SD 0:32 min:s). The total time to complete 37 navigation-related tasks ranged from a minimum of 3 min 57 s to a maximum of 32 min 42 s. Regression analysis showed that it took 6 s per click or step to successfully complete a task. To understand the nurses’ thought processes during monitor navigation, the authors analyzed the paths of the 2 tasks with the lowest successful completion rates, where only 13% (4/30) of the nurses correctly completed these 2 tasks. Although 30% (9/30) of the nurses accessed the correct screen first for task 1 and task 2, they could not find their way easily from there to successfully complete the 2 tasks.

**Conclusions:**

Usability testing of physiologic monitors revealed major ineffectiveness and inefficiencies in the current nurse-monitor interactions. The results indicate the potential for safety and productivity issues in completing routine tasks. Training on monitor use should include critical monitoring functions that are necessary for safe, effective, efficient, and appropriate monitoring to include knowledge of the shortest navigation path. It is imperative that vendors’ future monitor designs mimic clinicians’ thought processes for successful, safe, and efficient monitor navigation.

## Introduction

### Background

Closely observing the physiologic condition of critically ill patients is an essential and complex task that involves the use of sophisticated, computerized, and alarm-equipped physiologic monitors [1]. Research shows that an excessive number of false alarms (86%-99.5%) from physiologic monitors leads to a phenomenon called alarm fatigue [2-8], which further results in nurses having to respond to an average of 150-400 alarms per patient per day in intensive care units (ICUs) [9] and, more startlingly, ignoring alarms or inappropriately turning off alarms [10]. The Joint Commission and the Food and Drug Administration (FDA) attributed fatal alarm-related incidents to alarm fatigue [11,12]. As a result, The Joint Commission’s 2014 National Patient Safety Goal NPSG.06.01.01 mandated improving the safety of clinical alarm systems [13]. This study examined critical patient safety and usability problems related to physiologic monitors used by nurses in 4 adult ICUs. This study describes physiologic monitor use and alarm management effectiveness and efficiency—two goals of usability [14-16]. Self-perceived competence and nurse satisfaction with the use of monitors—a third goal of usability—was described elsewhere [17].

### Gap in Knowledge

Physiologic monitors, mostly heavily used by nurses, were associated with the highest number of alarms and alarms-related deaths in the FDA’s Manufacturer and User Facility Device Experience database and in previous research studies [12]. However, research involving nurses’ use of physiologic monitors is rare. The identification of critical usability issues for monitors, especially those related to patient safety, is a nursing imperative. The poor usability of physiologic monitors was one of the main themes identified by nurses in a recent study where changes in default alarm settings and standardized in-service education on monitor use were insufficient to improve the safety of the alarm system [10]. Nurses stated that the complexity of navigating monitors to manage parameters and alarm settings negatively affects the appropriate management of clinical alarms, threatens the timely recognition and response to lethal alarms, and induces high levels of frustration and unsafe workarounds among nurses [10]. Usability is a national priority for health care software. The 2012 Institute of Medicine report “Health Information Technology and Patient Safety: Building Safer Systems for Better Care” identified software usability as a critical attribute for patient safety [18]. However, little information is available about medical device usability, especially for nurses and specifically for clinical alarms management.

### Study Aims

The specific aims of this simulation-based usability study are directed toward the effectiveness and efficiency of bedside physiologic monitors and alarm management. The aims are consistent with usability attributes identified by the Institute of Medicine and human factor and usability engineering frameworks [14,15,18]. During observations of ICU nurses’ interactions with bedside physiologic monitors in a simulated environment, the study aims to: (1) examine successful task completion in the navigation of monitors (effectiveness), (2) examine the nurses’ thought processes during monitor navigation (effectiveness), (3) calculate the time required by nurses to navigate the monitors to perform different tasks (efficiency), and (4) calculate the number of clicks or steps it requires nurses to complete a monitor navigation task (efficiency).

## Methods

### Setting, Design, and Sample

The target units of this usability study were adult ICUs at a 705-bed university teaching hospital in the southwestern part of the United States. The ICUs were transplant and cardiac (37 nurses, 26 beds), surgical and trauma (55 nurses, 30 beds), neuro (28 nurses, 26 beds), and medical (53 nurses, 26 beds) units. The 4 ICUs have an annual admission rate of 5000 patients. After the approval by the institutional review board and following the recommendations by Faulkner for sample size in usability research [19], this interventional study used a convenience sample of 30 nurses from all the 4 ICUs. The study was conducted in a simulated environment using one of the ICU beds, a case scenario, a Philips IntelliVue MX800 bedside monitor, and a Philips IntelliVue Information Center iX central station monitor. The monitors are currently used in all the ICUs and have complex information systems that are capable of capturing, displaying, and storing waveforms; parameters and alarms; and include many menus, buttons, and icons for user navigation.

### Description of Tasks

The usability testing methods described here are congruent with the widely accepted usability techniques related to user tasks and outcome measures [14-16,18]. The principal investigator of the study and 3 expert ICU nurse educators created a short case scenario followed by 12 updates and/or changes in the patient’s medical condition and asked nurses to complete 40 tasks. Each update and/or a change in the patient’s medical condition was followed by a set of specific tasks. The case scenario, updates and/or changes in the patient’s medical condition, and the associated tasks were evaluated for face validity by 3 expert ICU nurses who assessed the appropriateness and complexity of tasks using a checklist and were piloted in the simulation environment for content, timing, and any methodological issues. Tasks were typical across all the ICUs and represent common monitoring tasks that are critical for appropriate monitoring and safe alarm management. Sowan and colleagues identified these tasks as a basic set of competencies for appropriate and safe monitoring operations [17]. The tasks targeted the following competencies and navigation actions [17]:

1. Admit, transport, and discharge patients using the monitors. Patient information needs to be correctly entered into the monitor for it to select the appropriate algorithm and calculate hemodynamic, oxygenation, and ventilation parameters for safe alarm limits. Nurses also need to know how to connect the monitor’s cables for multiparameter monitoring when a patient is admitted. The case scenario included 7 tasks in this category.
2. Manage measurements and monitor’s settings. After setting up the monitor and admitting a patient to the monitor, most of the nurses’ time is usually directed toward managing measurements and monitor’s settings. Examples include selecting the appropriate parameters for the patient condition, customizing measurement, and setting alarm limits to patient specific (ie, deactivating unnecessary parameters, setting the appropriate paced mode), adjusting the alarm volume and screen brightness, and adjusting the speed and size of the waves. The case scenario included 23 tasks.
3. Perform electrocardiogram (ECG) analysis. Performing a 12-lead ECG includes entering an order into the monitor, storing and sending the 12-lead ECG to the central monitor, and exporting the 12-lead ECG to the cardiology management system. The case scenario included 7 tasks related to the competency of analyzing the ECG.
4. Troubleshoot alarm conditions. Nurses are expected to troubleshoot common technical alarms (such as a lead-off alarm) and to follow the unit policy when they are troubleshooting alarms. The case scenario included 3 of these tasks.

### Study Procedure

Participation sessions were scheduled individually and video-recorded. Two expert nurse educators served as the moderators for the testing sessions and prepared the monitors based on the case scenario and tasks. Upon arrival, each participating nurse received a testing packet with a unique ID. The packet included directions for participation, a demographic form, the case scenario, and the tasks nurses need to execute using the monitors. Updates and changes in the patient medical condition in the testing packets were presented in a random order with the associated tasks on a separate page. Nurses were directed to complete the tasks in the order received and to think aloud for 3 tasks where no monitor-nurse interaction was possible (mentioned below). Nurses were asked to complete all the tasks, including those they did not know how to perform and indicate whether and when they would like to give up trying to perform a task. The moderator guided the nurse through the testing process, reminded the participant to think aloud when necessary, video-recorded the testing session, and printed the reports of the monitor settings before and after participation.

Because, in real life, nurses use the bedside monitors to set parameters and manage alarms, in this simulation study, efficiency and effectiveness of task completion were based on navigating the bedside monitor. The central station monitor was used to print reports of the settings of the bedside monitor pre- and postparticipation to measure the effectiveness of task completion.

### Outcome Measurements

The main outcome measures were effectiveness and efficiency in monitor use. Effectiveness was related to the success of completing the tasks in the case scenario and understanding the thought processes for task completion. Efficiency was concerned with the time of task completion and the number of clicks/steps taken for task completion.

#### Effectiveness

The principal investigator and 2 expert ICU nurse educators viewed all the videos and identified, classified, and validated successful task completion. The reports of parameters and alarm limits that were printed by the moderator from the central station monitor before and after each testing session were also used to validate the changes made by the participating nurse while judging the success of task completion. Furthermore, the nurse’s inability to complete a task was recorded as an unsuccessful completion of a task.

#### Efficiency

Efficiency was measured by the time of task completion (aim 2) and the number of clicks/steps taken for task completion (aim 3). Different screens and paths of navigation within the monitor are available to allow nurses to interact with the monitor. Nurses are expected to always select a short navigation path for task completion to enhance productivity and response to alarms. Understanding the navigation path of software is critical for identifying factors that may contribute to errors, efficiency, and catastrophic usability problems (eg, lack of responsiveness of the monitor to the change intended by nurses). The principal investigator and 2 expert ICU nurse educators viewed all the videos for the recorded start and end times for each task and determined efficient pathways for task completion. The time for each task started from the time the nurse started interacting with the monitor. In total, 3 of the 40 tasks did not require monitor navigation; therefore, efficiency was limited to 37 navigation-related tasks.

### Data Analysis

Descriptive statistics were used to describe the sample characteristics and main study outcomes. The success of task completion and time were presented for each task. Simple regression analysis was used to measure the association between the number of clicks/steps taken per task and time in seconds for task completion.

## Results

### Nurse Characteristics

A total of 30 nurses participated in the simulation study. The majority of the nurses were from neuro ICU (14/30, 47%) and surgical trauma ICU (11/30, 37%), females (25/30, 83%), full-time employees (18/30, 60%), with less than 3 years of experience in their ICU (19/30, 63%), had 3 or more years working as a nurse (17/30, 57%), and had not received training on the monitors within the last 2 months (25/30, 83%).

### Effectiveness of Task Completion

Among the 40 tasks, only 2 (5%) were completed correctly by all the 30 nurses (ie, “take the monitor out of standby” and “verify noninvasive blood pressure [NBP] is set to every 15 min”). At least one (1/30, 3%) nurse abandoned or did not successfully perform 38 tasks. In total, 50% (15/30) to 90% (27/30) of the nurses could not successfully complete 8 tasks. The tasks with the lowest successful completion rates were “explain how to change resuscitation status in the monitor” (completion rate was 3/30, 10% nurses;); “adjust screen brightness to 7” (4/30, 13% nurses); “record a 25 mm/s ECG strip of any of the ECG leads” (4/30, 13% nurses); “troubleshoot the source of alarm by making sure X2 (transport monitor) is synched correctly to bedside monitor” (6/30, 20%); “verify the source of alarm is from MAP (mean arterial pressure) and systolic and change source if needed” (9/30, 30%); “disconnect the X2 and place bedside monitor on standby” (12/30, 40%); “troubleshoot false ECG alarms on the monitor” (15/30, 50%); and “change NBP MAP lower limit to 65” (15/30, 50%).

To understand the nurses’ thought processes during monitor navigation, the authors analyzed the paths of the 2 tasks with the lowest successful completion rates, where only 13% (4/30) of the nurses completed correctly: “record a 25 mm/s ECG strip of any of the ECG leads” and “adjust screen brightness to 7.” Multimedia Appendix 1 shows 2 correct paths followed by nurses who took 6 and 7 steps to “record a 25 mm/s ECG strip of any of the ECG leads.” Based on the correct paths, the key steps for task completion were *taskbar* and *recording.* Multimedia Appendix 1 also shows the analysis of the first 3 steps performed by the nurses who unsuccessfully completed the task. For example, 27% (8/30) nurses started the task by accessing the *xheart rate (HR) waveform* screen, 20% (6/30) started by *taskbar*, and 10% (3/30) started by *HR numeric*. The most common second step (15/30, 50% of the nurses) was scrolling back and forth for 1, 2, 3, or 4 times. It appears that these nurses were trying to find the best screen to click next to record a 25 mm/s ECG strip. Some nurses also accessed *12-lead*, *capture 12-lead*, *capture ECG*, and *setup ECG* as a second step (5/30, 17%). *Capture 12-lead* and *setup ECG* were also clicked by 33% (10/30) nurses as a third step for task completion.

Similarly, for “adjust screen brightness,” the key screen for a successful path was *user interface*, which can be accessed after the *main setup screen*. Although 30% (9/30) of the nurses who unsuccessfully completed this task accessed the *main setup* first, they unsuccessfully scrolled back and forth or accessed *equipment* as their second step to change the screen brightness. The *main setup* was accessed in the first (9/30, 30%), second (2/30, 6%), and third (2/30, 6%) steps by nurses, but it appears that nurses did not know what to click next to adjust brightness.

### Efficiency in Task Completion

Efficiency analysis focused on the time to *successfully* complete the task because among nurses who could not successfully perform the task, some nurses gave up quickly, whereas others spent more time trying to complete a task. Tables 1-4 present the mean time and range (in min:s) for successful task completion. The tables include the time to complete 37 (vs 40) tasks. Time was not recorded for the following 3 tasks because they were explained by nurses during the simulation and they required no to minimal monitor-nurse interaction: (1) “what would you do if you had INOP (inoperative or technical) alarm and didn’t know how to troubleshoot it,” (2) “explain how to change the resuscitation status in the monitor,” and (3) “explain how to discharge a patient from the monitor.”

**Table 1:** Mean time for task completion (min:s) for admission, discharge, and transfer-related tasks (N=30 nurses).

Task	Task completion time		Successfully completed tasks, n (%)
	Mean (SD)	Range	
Take the monitor out of standby	0:02 (0:01)	0.01-0.08	30 (100)
Disconnect the X2^a^ and place bedside monitor on standby	0:08 (0:08)	0:02-0:59	12 (40)
Select the correct patient profile	0:11 (0:06)	0:02-0:32	22 (73)
Reconnect X2 and readmit the patient to the bedside monitor	0:20 (0:16)	0:05-1:15	26 (87)
Set up the cables for each A-line^b^ and CVP^c^	0:27 (0:17)	0:06-1:28	26 (87)
Admit the patient into the monitor	1:11 (0:29)	0:31-2:02	28 (94)

^a^X2: name of the transport monitor.

^b^A-line: arterial line.

^c^CVP: central venous pressure.

**Table 2:** Mean time for task completion (min:s) for managing measurements and monitor settings-related tasks (N=30 nurses).

Task	Task completion time		Successfully completed tasks, n (%)
	Mean (SD)	Range	
Adjust alarm volume to be quieter	0:03 (0:02)	0:02-0:09	23 (77)
Print parameters’ limits for all active alarms	0:04 (0:03)	0:01-0:15	27 (9)
Verify that NBP^a^ is set to q15 min	0:05 (0:03)	0:02-0:16	30 (100)
Pause ABP^b^/ART^c^ alarm while A-line^d^ is being inserted	0:06 (0:06)	0:01-0:30	24 (80)
Identify on the screen for how long the alarm will be paused	0:07 (0:06)	0:02-0:28	26 (87)
Deactivate ART alarm	0:09 (0:05)	0:04-0:30	28 (93)
Verify vitals are displayed: NBP, Temp^e^, RR^f^, SpO_2_^g^	0:09 (0:10)	0:02-0:53	26 (87)
Adjust RR waveform size up	0:10 (0:06)	0:05-0:33	24 (80)
Display the missing vitals	0:10 (0:09)	0:02-0:32	26 (87)
Turn on QRS^h^ volume on SpO_2_ and turn off the volume	0:13 (0:06)	0:06-0:33	26 (87)
View upper/lower limits of active parameters	0:13 (0:07)	0:03-0:36	27 (90)
Adjust alarm volume to be louder	0:13 (0:11)	0:02-0:42	25 (83)
Change wave speed on SpO_2_ to be faster	0:14 (0:09)	0:02-0:37	19 (63)
Change paced mode to off	0:14 (0:20)	0:01-1:27	19 (63)
Change upper and lower values of heart rhythms	0:15 (0:06)	0:06-0:40	29 (97)
Change upper or lower blood pressure limits to patient specific	0:23 (0:09)	0:11-0:52	27 (90)
Verify source of alarm is from MAP^i^ and systolic	0:26 (0:26)	0:07-1:25	9 (30)
Change NBP MAP lower limit to 65	0:30 (0:28)	0:03-1:26	15 (50)
Adjust screen's brightness up to 7	0:37 (0:12)	0:28-0:54	4 (13)
On X2^j^, change SpO_2_ to Res^k^	0:38 (0:19)	0:13-1:27	17 (57)
Verify waveforms for A-line/CVP^l^ parameters are displayed	0:38 (0:55)	0:04-4:03	22 (74)
Turn A-fib^m^ and irregular HR^n^ to off	0:42 (0:18)	0:20-1:26	16 (54)

^a^NBP: noninvasive blood pressure.

^b^ABP: arterial blood pressure.

^c^ART: alternative arterial.

^d^A-line: arterial line.

^e^Temp: temperature.

^f^RR: respiratory rate.

^g^SpO_2_: peripheral capillary oxygen saturation.

^h^QRS: a name of a wave in the electrocardiogram.

^i^MAP: mean arterial pressure.

^j^X2: name of the transport monitor.

^k^Res: respiration.

^l^CVP: central venous pressure.

^m^A-fib: atrial fibrillation.

^n^HR: heart rate.

**Table 3:** Mean time for task completion (min:s) for performing electrocardiogram analysis–related tasks (N=30 nurses).

Task	Task completion time			Successfully completed tasks, n (%)
	Mean (SD)	Range		
Print ECG^a^ report	0:03 (0:02)	0:01-0:15	27 (90)	
Export ECG	0:04 (0:03)	0:01-0:21	27 (90)	
Switch the primary lead to lead III	0:08 (0:04)	0:04-0:27	29 (97)	
Perform 12-lead ECG, enter the order #	0:08 (0:08)	0:11-1:25	18 (60)	
Show ECG analysis	0:10 (0:06)	0:01-0:09	20 (67)	
Store and send ECG analysis	0:31 (0:17)	0:01-0:12	28 (93)	
Record a 25 mm/s ECG strip of any of the ECG leads	1:14 (0:32)	0:27-1:39	4 (13)	

^a^ECG: electrocardiogram.

**Table 4:** Mean time for task completion (min:s) for performing troubleshooting alarms-related tasks (N=30 nurses).

Task	Task completion time			Successfully completed tasks, n (%)
	Mean (SD)	Range		
Troubleshoot false ECG^a^ alarms on the monitor	0:21 (0:16)	0:03-0:53	15 (50)	
Troubleshoot source of alarm by making sure X2^b^ is synched to bedside	0:29 (0:14)	0:14-0:54	6 (20)	

^a^ECG: electrocardiogram.

^b^X2: name of the transport monitor.

The task “Take monitor out of standby” had the shortest completion time (Table 1, mean 0:02, SD 0:01 min:s), whereas the task “record a 25 mm/s ECG strip of any of the ECG leads” had the longest completion time (Table 3, mean 1:14, SD 0:32 min:s). The task completion time range in Tables 1-4 provides valuable information about the variation in time it took nurses to successfully complete the tasks. For example, although the task “verify waveforms for A-line (arterial line) or CVP (central venous pressure) parameters are displayed” took nurses an average of 38 s to successfully complete it (mean 0:38, SD 0:55 min:s), some nurses spent only 4 s to complete this task, whereas other nurses spent as long as 4 min to complete it (Table 2). Across nurses, the minimum total time for task completion for all 37 tasks was 237 s (3 min 57 s), whereas the maximum was 1962 s (32 min 42 s).

**Figure 1:**
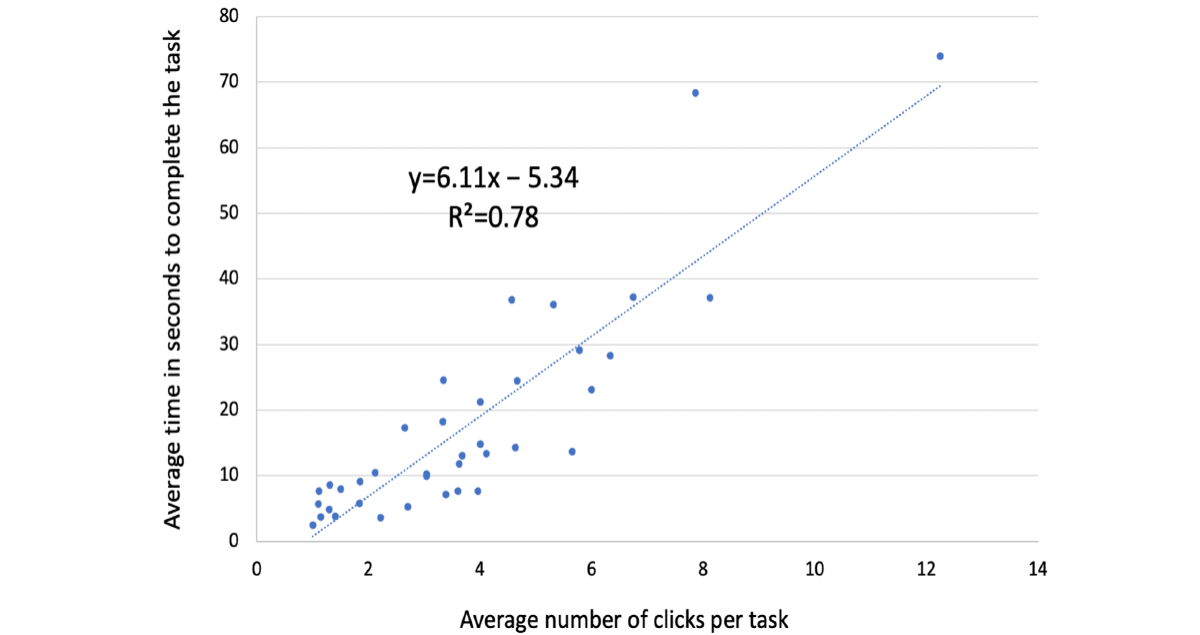
Regression analysis of mean number of clicks taken per task and average time in seconds for a successful task completion (N=37 tasks).

A linear regression analysis (Figure 1) of mean successfully completed tasks (N=37) revealed that it took nurses 6.11 s per additional click (or a step) on the monitor to perform a task during monitor navigation (*y*=6.11−5.34, *R^2^*=0.78, *P*=.001).

Among task completers, some nurses completed a task in the first attempt, whereas other nurses took more than one attempt. Nurses who completed the tasks in the first attempt took an average of 3.5 clicks (or steps) and 16.5 s per task as compared with 8 clicks and 35 s per task for those who completed the tasks in more than one attempt.

## Discussion

### Principal Findings

The past few years have witnessed a growing number of quality improvement and interventional research studies directed toward reducing alarm fatigue and improving alarm system safety [10,20,21]. Efforts focused on pulse oximetry and physiologic monitors, including tight versus loose peripheral capillary oxygen saturation alarm strategy [20], patient-customized monitoring bundles and thresholds [10,21-23], nurse education [10], and utilization of patient profiles and updated bedside visual reminders [23]. Although these efforts led to a significant reduction in the number of nuisance alarms, the reduction was insufficient to improve nurses’ attitudes toward alarms or their perceptions of alarm fatigue in ICUs [10]. The complexity of modern alarm devices requires usability testing for a safe and efficient operation of medical devices [24]. To our knowledge, this is the first study to examine the usability of physiologic monitors, the number one device associated with sentinel events in the FDA database, and the one with the highest number of nonactionable alarms [11,12].

This study examined nurses’ effectiveness and efficiency in completing 40 common tasks as nurses interacted with bedside physiologic monitors. The results indicate the potential for continued safety issues in completing the routine monitoring tasks. Not a single nurse performed all the tasks correctly, and some performed more than one incorrectly. Surprisingly and perhaps even startlingly, many of these tasks represent routine everyday-monitoring tasks such as, “verify certain vitals are displayed on the monitor,” “view upper and lower limits of all parameters,” “display missing vitals in the monitor,” “print alarm parameters’ limits,” “verify that NBP is set to q 15 min,” and “set up the cables for each A-line and CVP.” Other tasks were critical to customizing parameters to be patient specific, individualizing the monitoring process, eliminating over-and undermonitoring, decreasing the number of unnecessary alarms, and thereby improving nurse safety and productivity in monitoring and decreasing alarm fatigue. Examples of these tasks are “change paced mode to off,” “switch the primary lead to lead III,” “change upper and lower heart rhythms,” “select the correct patient profile,” “change upper and lower blood pressure limits to patient specific,” “turn atrial fibrillation and Irregular HR to off,” “pause the ABP (arterial blood pressure)/ART (alternative arterial) alarm while the line is being inserted,” “change NBP MAP lower limit to 65 on the monitor,” and “deactivate ART parameter.”

Some nurses were also unable to “admit the patient into the monitor,” “reconnect X2 and readmit patient to bedside monitor,” “take monitor out of standby,” and to “explain how to discharge a patient from the monitor.” However, it is important to note that these skills are context-specific. For example, when this study was conducted, part of a nurse’s job was to admit the patient into the monitor. This process was recently streamlined, and patients are now admitted into the monitor via our admission, discharge, and transfer department. Similarly, all monitors are set to brightness level 5. Tasks such as “adjust brightness of screen up to 7” might not be as frequently used as other tasks; however, this is an important design feature for screen visibility. In addition, the monitor allows nurses to adjust the alarm volume, which is a critical function to provide a quieter care environment, specifically during the night shift, and improve patients’ Hospital Consumer Assessment of Healthcare Providers and Systems scores. However, 10 (out of 30) of our nurses were unable to perform this task.

Regarding efficiency, there was a 30-min difference between the shortest and longest times to correctly perform the 37 navigation-related tasks. The monitor allows nurses to perform tasks using different navigation paths. Some paths are shorter than others, but both types of paths are rated as correct in a successful task completion. For example, a nurse admitted a patient to the monitor using the following 5-click (or steps) navigation path and completed the task in 31 s: “(1) patient demographics, (2) admit patient, (3) MRN (medical record number), (4) VIN # (visit identification number), and (5) confirm,” whereas another nurse took 2 min 2 s to complete the same task following a longer 11-click navigation path: “(1) main setup, (2) taskbar, (3) arrow X1, (4) patient demographics, (5) MRN #, (6) VIN #, (7) confirm, (8) last name, (9) first name, (10) confirm, and finally (11) main screen.” Regression analysis revealed that nurses took 6.11 s per additional click (or step) on the monitor to successfully perform a task during monitor navigation. Our results also suggest that training nurses on the shortest navigation paths could save up to 30 min per nurse to complete the 37 routine monitor navigation tasks examined here.

The results also showed that some nurses followed a definitive short path in correctly performing a task, whereas other nurses successfully completed a task using multiple attempts, may be as a “trial and error.” For example, one nurse followed the following path to “change paced mode to off” and completed the task in 2 s and 3 clicks: “(1) patient demographics, (2) paced mode, and (3) off,” whereas another nurse used trial and error to complete the same task as evidenced by entering and exiting multiple screens searching for the parameter to be changed and managed to complete the task in 27 s and 10 clicks: “(1) HR waveform, (2) *exit*, (3) main setup, (4) equipment, (5) *exit*, (6) main setup, (7) *exit*, (8) measurement, (9) paced mode, and (10) off.” Nurses who successfully completed the tasks but performed more than one attempt used on an average 5 extra clicks per task and 18 extra s per task compared with those who completed the tasks at the first attempt. These results reflect a lack of familiarity with the task and the most efficient navigation path in the monitor to complete tasks. This provides further evidence for the need for detailed training on monitor use.

Nurse-monitor navigation is a complex cognitive process that requires adherence to policies and procedures, a usable monitor design, sufficient training on monitor functions, and the use of clinical reasoning for appropriate monitoring to eliminate over- or undermonitoring. Understanding this cognitive process is critical for safe and appropriate monitoring. For example, all the nurses who were unable to successfully complete “pause the ABP/ART alarm while the line is being inserted” task navigated to *setup ABP* or *ABP numeric* to complete the task. It appears that nurses were expecting to complete the task from the accessed screens (*setup ABP* or *ABP numeric*). This result demonstrates the importance of designing a monitor’s functions in a way that mimics clinicians’ thought processes for a successful navigation. Analyzing the paths of the two tasks with the lowest successful completion rates supported these results. For example, although almost half of the nurses accessed the *main setup* during monitor navigation to adjust the screen brightness, none of these nurses accessed *user interface* as a subsequent step. Supporting the fact that nurses did not think that brightness can be found under *user interface* screen. Similarly, to record an ECG strip, many of the nurses navigated *12-lead*, *capture 12-lead*, *capture ECG*, or *setup ECG* screens instead of *taskbar* or *HR numeric* screens. In fact, it makes sense to complete such a task under the screens visited by nurses.

### Limitations

The study results should be interpreted considering the following limitations. First, the study included a sample size appropriate for usability studies. Nevertheless, the sample size was only 17.3% (30/173) of the 173 ICU nurses in all the 4 adult ICUs. Including a stratified sample representing all ICUs could improve the generalizability of the study. A criticism might be that the convenience sample resulted in nurses with slower efficiency time to participate in the study. However, we contend that nurses who were not confident in using monitors would not have self-selected to be in this study. A stratified sample might cause even more variable efficiency and effectiveness results. Second, monitoring policies are context-sensitive. For example, in some hospitals, customizing parameters and alarm limits to patient-specific ones is the job of a physician and not a bedside nurse. Adherence to monitoring policy within a specific context is important for a valid usability test; however, it may limit the generalizability of the study. Third, vendors may have their own terminologies built into their particular monitors. For example, the term INOP alarm is specific to Philips monitors and not applicable to the General Electric monitors. Replicating this study in other hospitals would require the use of appropriate terminologies that are applicable to the medical device under study. Fourth, this study examined 40 common nurse-monitor navigation tasks. The rapid advancements in technology may eliminate some of these tasks or add to the list of tasks that nurses can perform using the monitors in the future. Future researchers will want to reassess the task lists.

### Conclusions

Usability testing of physiologic monitors in this setting revealed major ineffectiveness and inefficiencies in nurse-monitor interactions. The results have implications for both safety and productivity. Training on monitor use should include critical monitoring tasks and functions that are necessary for safe and appropriate monitoring as well as the shortest path to navigate the monitor to increase nurse productivity and response to alarms. An imperative is for vendors to design the monitoring functions to mimic clinicians’ thought processes for a successful, safe, and efficient monitor navigation.

## Appendix

Multimedia Appendix 1 Analysis of nurses’ thought processes during task completion.

## Abbreviations

ABP: arterial blood pressure
A-line: arterial line
ART: alternative arterial
CVP: central venous pressure
ECG: electrocardiogram
FDA: Food and Drug Administration
HR: heart rate
ICU: intensive care unit
INOP: inoperative or technical
MAP: mean arterial pressure
MRN: medical record number
NBP: noninvasive blood pressure
VIN: visit identification number
X2: name of the transport monitor
