# Supplementary material for: Improving the Safety, Effectiveness, and Efficiency of Clinical Alarm Systems: Simulation-Based Usability Testing of Physiologic Monitors
Source: JMIR Nurs. 2021 Feb 3;4(1):e20584. doi: 10.2196/20584 (PMC8328265; doi:10.2196/20584)
Supplement: Multimedia Appendix 1 [file nursing_v4i1e20584_app1.docx]

**Multimedia Appendix 1. Analysis of nurses’ thought processes during task completion (N=30).**

| **Correct paths to “record a 25mm/sec ECG^a^ strip of any of the ECG leads”** | | | | | | |
| --- | --- | --- | --- | --- | --- | --- |
| **Step 1** | **Step 2** | **Step 3** | **Step 4** | **Step 5** | **Step 6** | **Step 7** |
| Taskbar | Scrolled×1 | Recordings | Primary lead | 25 mm | Confirm | N/A^b^ |
| HR^c^ numeric | Task bar | Start/stop recording | Wave selection | Lead II | Confirm | 25 mm/s |
| **First 3 steps of the incorrect paths to “record a 25mm/sec ECG strip of any of the ECG leads”** | | | | | | |
| **Step 1** | **Frequency (%)** | **Step 2** | **Frequency (%)** | **Step 3** | **Frequency (%)** | N/A |
| HR waveform | 8 (27) | Scrolled×1, 2, 3, 4 | 15 (50) | Capture 12-lead | 7 (23) | N/A |
| Taskbar | 6 (20) | 12 lead | 2 (6) | Setup ECGa | 3 (10) | N/A |
| HR numeric | 3 (10) | Capture 12-lead | 1 (3) | Scrolled×2 | 2 (6) | N/A |
| ECG wave | 3 (10) | Capture ECGa | 1 (3) | Capture waves | 2 (6) | N/A |
| Set up ECG | 2 (6) | Setup ECGa | 1 (3) | HR waveform | 2 (6) | N/A |
| N/A | N/A | Print reports | 1 (3) | N/A | N/A | N/A |
| **Correct pats to “adjust screen brightness up to 7”** | | | | | | |
| **Step 1** | **Step 2** | **Step 3** | **Step 4** | **Step 5** | **N/A** | **N/A** |
| Main setup | scroll×2 | user interface | display 1 | brightness | N/A | N/A |
| **First 3 steps of the incorrect paths to “adjust screen brightness up to 7”** | | | | | | |
| **Step 1** | **Frequency (%)** | **Step 2** | **Frequency (%)** | **Step 3** | **Frequency (%)** | N/A |
| Main setup | 9 (30) | Scroll×1, 2, 3, 4, 6 | 13 (43) | Exit | 6 (20) | N/A |
| Change screen | 5 (17) | Equipment | 3 (10) | Task bar | 3 (10) | N/A |
| Task bar | 4 (13) | Main screen | 2 (6) | Alarm volume | 3 (10) | N/A |
| Main screen | 2 | Main setup | 2 (6) | Main setup | 2 (6) | N/A |
| N/A | N/A | Change screen | 1 (3) | Main screen | 1 (3) | N/A |
| N/A | N/A | Taskbar | 1 (3) | Change screen | 1 (3) | N/A |

^a^ECG: electrocardiogram.

^b^N/A: not applicable.

^c^HR: heart rate.
